# Transcriptome Analysis of Ethylene-Related Genes in Chlorine Dioxide-Treated Fresh-Cut Cauliflower

**DOI:** 10.3390/genes15081102

**Published:** 2024-08-21

**Authors:** Weiwei Jin, Qiaojun Jiang, Haijun Zhao, Fengxian Su, Yan Li, Shaolan Yang

**Affiliations:** 1 Institute of Food Science, Wenzhou Academy of Agricultural Science, Wenzhou 325006, China; wwjin2003@163.com (W.J.); jiangqiaojun7432@126.com (Q.J.); 18293997473@163.com (H.Z.); sufengxian@wzvcst.edu.cn (F.S.); 2 Southern Zhejiang Key Laboratory of Crop Breeding, Wenzhou 325006, China; 3 College of Life Science and Engineering, Lanzhou University of Technology, Lanzhou 730050, China; 4 College of Horticulture, Qingdao Agricultural University, Qingdao 266109, China; shaolanyang@126.com

**Keywords:** fresh-cut cauliflower, chlorine dioxide, transcriptome, RNA-seq, ethylene, stress

## Abstract

Chlorine dioxide (ClO_2_) is widely used for the quality preservation of postharvest horticultural plants. However, the molecular mechanism of how ClO_2_ works is not clear. The purpose of this study was to understand ethylene-related molecular signaling in ClO_2_-treated fresh-cut cauliflower florets. Transcriptome analysis was used to investigate ethylene-related gene regulation. A total of 182.83 Gb clean data were acquired, and the reads of each sample to the unique mapped position of the reference genome could reach more than 85.51%. A sum of 2875, 3500, 4582 and 1906 differential expressed genes (DEGs) were identified at 0 d, 4 d, 8 d and 16 d between the control group and ClO_2_-treated group, respectively. DEGs were enriched in functions such as ‘response to oxygen-containing compounds’ and ‘phosphorylation’, as well as MAPK signaling pathway, plant hormone transduction pathway and so on. Genes, including *OXI1*, *MPK3*, *WRKY22* and *ERF1*, which are located at the junction of wounding, pathogen attack, pathogen infection or ethylene signal transduction pathways, were up-regulated in response to stress. *ETR* and *CTR1* (both up-regulated), as well as three down-regulated genes, including *BolC5t34953H* (a probable *NAC*), *BolC1t05767H* (a probable *NAC*) and *BolC2t06548H* (a probable *ERF13*), might work as negative regulators for ethylene signal transduction. In conclusion, ethylene-related genes and pathways are involved in ClO_2_ treatment, which might enhance stress resistance and have a negative feedback mechanism.

## 1. Introduction

Cruciferae vegetables are widely cultivated in the world. They are rich in nutrition and health-promoting components, e.g., glucosinolates and isothiocyanates, which may help reduce the risk of cancer [1,2,3,4]. Currently, fresh-cut vegetables have become more popular among consumers because of their convenience [5]. Cauliflower (*Brassica oleracea* L. *var. botrytis*), as one of the largest varieties of Cruciferae vegetables, patently accounts for a large proportion of the ready-to-eat market. However, fresh-cut cauliflower is more susceptible to microbes due to mechanical injury, resulting in faster quality deterioration, including senescence, mildew and decay [6]. Lots of treatments, such as ultrasound, gamma irradiation, electrostatic field and packaging, have been applied to fresh-cut cauliflower for quality maintenance and shelf-life extending [6,7,8,9,10]. But there are few reports on the effect of chlorine dioxide (ClO_2_) treatment on the preservation of fresh-cut cauliflower.

ClO_2_ has been used as a Class A1 disinfectant for food preservation around the world [11,12]. The broad antimicrobial effect of ClO_2_ on bacteria, fungi, spores and virus is well known, as well as the higher oxidative capacity with the lower concentration and shorter treatment time of ClO_2_ compared to chlorine [13]. A treatment of 60 mg·L^−1^ ClO_2_ for 10 min on fresh-cut coriander could decrease the total number of aerobic bacterial colonies to 2.1 lg CFU/g [14]. In addition to sterilization, ClO_2_ shows superiority for the postharvest storage of horticultural produce [15]. A treatment of 100 mg·L^−1^ ClO_2_ for 20 min could inhibit enzymatic browning and prolong the shelf-life of fresh-cut asparagus lettuce to 14 d [16]. The activities of enzymes related to senescence and lignification in fresh-cut bamboo were effectively inhibited after treatment with 28 mg·L^−1^ ClO_2_ plus chitosan coating [17]. Lin et al. found by transcriptome analysis that 50 mg·L^−1^ ClO_2_ could effectively keep the floret green and delay the senescence process of fresh-cut broccoli [18].

As a kind of phytohormone, ethylene is known for its role in the ripening of horticultural plants, eventually leading to senescence [19]. In addition, ethylene also plays a significant role in pathogens, heat and cold stress responses [20,21,22,23]. Lin et al. reported that ClO_2_ treatment could inhibit ethylene biosynthesis through the regulation of ethylene-responsive transcription factor (*ERF*) expression and delay the yellowing of fresh-cut broccoli [18]. But how ClO_2_ treatment affects ethylene-related gene expression in fresh-cut cauliflower remains unknown.

To reveal the effect of ClO_2_ treatment on fresh-cut cauliflower at the molecular level, a transcriptome analysis of ethylene-related genes in fresh-cut cauliflower florets regulated by ClO_2_ treatment was carried out in this study. DEGs regulated by ClO_2_ treatment were screened out, and the signaling pathways involved in DEGs were analyzed. This study may enrich comprehension and lay the foundation for further research on the function mechanism of ClO_2_ treatment.

## 2. Materials and Methods

### 2.1. Experimental Materials and Treatment Methods

Fresh white cauliflowers (*Brassica oleracea L. var. botrytis*) were harvested from the farm of Wenzhou Academy of Agricultural Sciences and were transported directly to Southern Zhejiang Key Laboratory of Crop Breeding within 1 h. Cauliflowers were selected for uniformity of size, weight and absence of any defect, mechanical injury or decay. After manual removal of outer leaves, cauliflower was cut into individual florets of 4–5 cm length. The cutting board, knife and hands were sterilized with 50 mg·L^−1^ sodium hypochlorite solution (pH 6.5).

The cauliflower florets were divided randomly into two groups and were immersed in deionized water (the control group, CK) or 100 mg·L^−1^ chlorine dioxide solutions (the treatment group, T) for 15 min and then air-dried. Subsequently, each group of florets weighting approximately 200 g was packaged in a polyethylene film bag (thickness: 6 μm, dimensions = 40 cm × 35 cm) and then stored under 4 °C ± 1 and 85 ± 5% RH in a refrigerator. Samples were collected at 0, 4, 8 and 16 d after cold storage and frozen in liquid nitrogen and stored at −80 °C for further study. Three biological replicates of each sample were used in the experiments.

### 2.2. RNA-Seq Analysis

The cauliflower floret samples were sent to Shanghai Majorbio Bio-pharm Technology Co., Ltd. (Shanghai, China) for RNA sequencing and bioinformatics analysis. Total RNA was extracted from the cauliflower florets. A quality evaluation of isolated RNA including purity and concentration, integrity and RNA quality number (RQN) was performed by NanoDrop 2000, agarose gel electrophoresis and Agilent 5300, respectively. Single cDNA library construction required that the total RNA of all samples was 1 μg, the concentration was greater than 30 ng·μL^−1^, RQN > 6.5, and OD_260_/OD_280_ was between 1.8 and 2.0.

The library preparation was sequenced on the Illumina NovaSeq X Plus platform. Fastp software (version 0.23.4, https://github.com/OpenGene/fastp, accessed on 5 July 2024) was used to filter out repetitive redundant sequences and low-quality sequences from the raw reads to obtain high-quality sequences (clean reads) for subsequent analysis. Reads were mapped to the *Boleracea* HDEM Genome database (https://www.genoscope.cns.fr/externe/plants/chromosomes.html, accessed on 5 July 2024) by using Hisat2 software (version 2.2.1, https://daehwankimlab.github.io/hisat2/, accessed on 5 July 2024). Stringtie software (version 2.2.1, https://ccb.jhu.edu/software/stringtie/, accessed on 5 July 2024) was used for reference-based assembly and quantification.

The expression levels of genes were calculated using FPKM (fragments per kilobase of transcript per million fragments mapped) with RSEM software (version 1.3.3, http://deweylab.biostat.wisc.edu/rsem/, accessed on 8 July 2024). DESeq2 software (version 1.42.0, https://bioconductor.org/packages/release/bioc/html/DESeq2.html, accessed on 8 July 2024) was used to standardize the read count of differential expressed genes (DEGs) between the CK group and T group of cauliflower florets. DEGs were screened by |log_2_ (fold change)| ≥ 1 and P_adj_ value < 0.05. BLAST+ software (version 2.9.0, https://ftp.ncbi.nlm.nih.gov/blast/executables/blast+/2.9.0/, accessed on 8 July 2024) was used to compare DEGs with Gene Ontology (GO) and KEGG (Kyoto Encyclopedia of Genes and Gnomes) databases. GO enrichment analysis of DEGs was performed using Goatools software (version 1.4.4, https://pypi.org/project/goatools/, accessed on 8 July 2024). A KEGG signaling pathway enrichment analysis of DEGs was performed using R script. Both analyses required P_adj_ value < 0.05 as the criterion for enrichment.

## 3. Results

### 3.1. Transcriptome Sequencing Quality Assessment

To explore the regulation of ethylene-related genes after ClO_2_ treatment, transcriptome analysis was performed on 24 cauliflower floret samples using RNA-Seq technology. 182.83 Gb of clean data were acquired, and each sample received approximately 6.53 Gb of clean data, with the proportion of Q30 bases exceeding 96.42%. The clean data of each sample were mapped to the reference genome for sequence comparison. The comparison rate ranged from 89.55% to 90.48%, and the reads of each sample to the unique mapped position of the reference genome could reach more than 85.51%. Descriptive statistics on the quality of the transcriptome sequencing of cauliflower florets treated with ClO_2_ are shown in Table 1.

**Figure 1:**
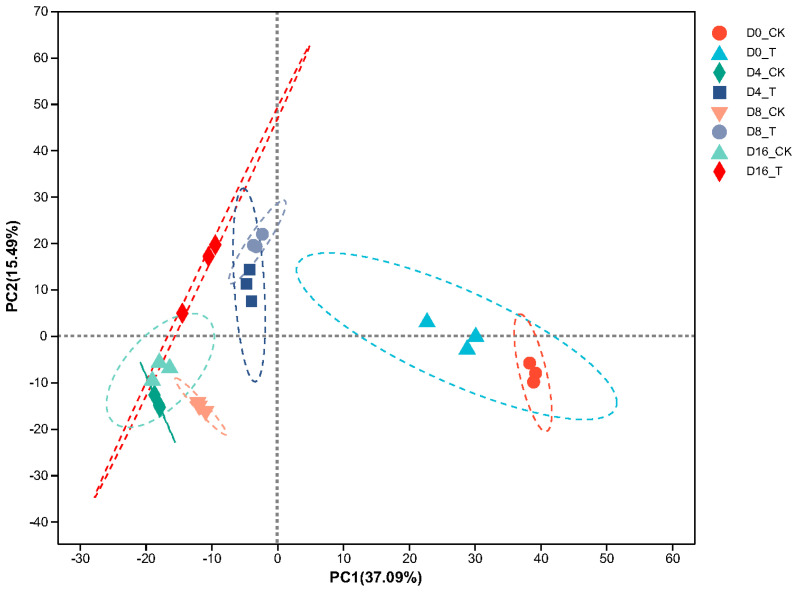
PCA analysis of transcriptome data in ClO_2_ (T) and control (CK) group during storage.

PCA calculation was executed on the transcriptome data to detect the similarity and variability between the ClO_2_ treatment (T) and the control (CK) groups. The results are shown in Figure 1. The principal component 1 (PC1) accounted for 37.09% of the variance and principal component 2 (PC2) accounted for 15.49% of the variance. Both at the level of PC1 and PC2, the T group samples at 4 d and 8 d were well separated from the CK group samples at the same time point, respectively. However, the differentiation between the T group and CK group at the early storage time (0 d) and the ending storage time (16 d) was less apparent. The results showed that clear separation between ClO_2_ treatment and control samples was observed during the middle storage period. The biological replicates were mainly grouped along PC2, which suggests larger variations between experimental treatments than biological replicates. And it is clear that samples at 0 d showed the greatest variation when compared with samples at other time points.

### 3.2. Identification and Analysis of DEGs

**Figure 2:**
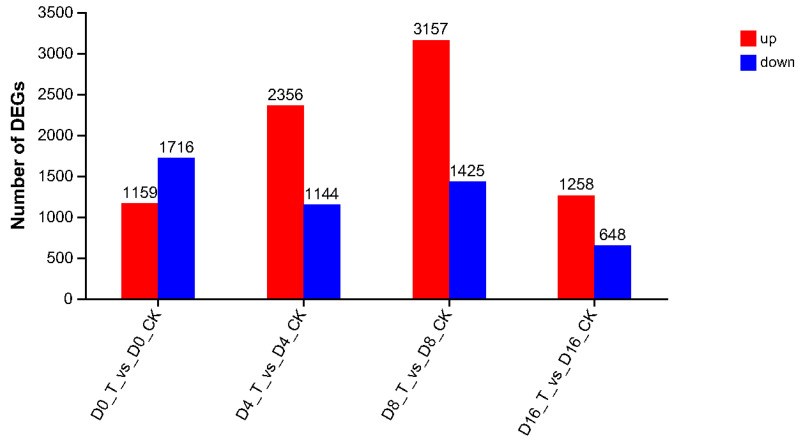
Analysis of DEGs in different comparison periods in ClO_2_ (T) and control (CK) group of fresh-cut cauliflower florets during storage.

The results of DEG screening from CK and T groups of cauliflower florets are shown in Figure 2. Fold change (FC) represents the ratio of gene expression between CK and T groups. When the value of |log_2_ (FC) | was greater than 1, the gene was identified as an up-regulated expression; otherwise, it was down-regulated.

The number of DEGs during the whole storage exhibited a pattern of initial increase followed by subsequent decrease. At 0 d, 1159 DEGs were up-regulated, and 1716 DEGs were down-regulated in the ClO_2_ group compared with the control. The numbers of up-regulated DEGs increased until 8 d and then declined significantly at 16 d, which was consistent with the trend of total DEGs. The results of DEGs between the ClO_2_ treatment and control groups were consistent with those of PCA analysis from Figure 1.

**Figure 3:**
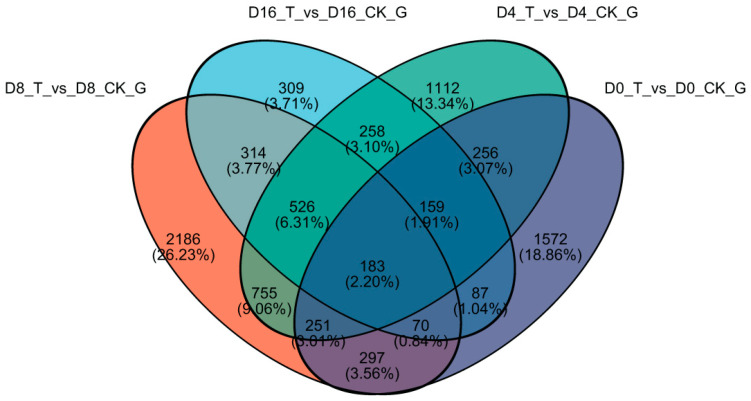
Venn diagram of DEGs in ClO_2_ (T) and control (CK) group of fresh-cut cauliflower florets during storage.

The Venn diagram of DEGs during storage is shown in Figure 3. Among all DEGs, 183 genes exhibited presence at every storage time in the ClO_2_ and control groups, and further study on the 183 genes was conducted subsequently. 499 DEGs were shared between D0_T vs D0_CK and D16_T vs D16_CK, which was the least number among the pair-wise comparison of all samples.

### 3.3. Go Function Analysis of DEGs

**Figure 4:**
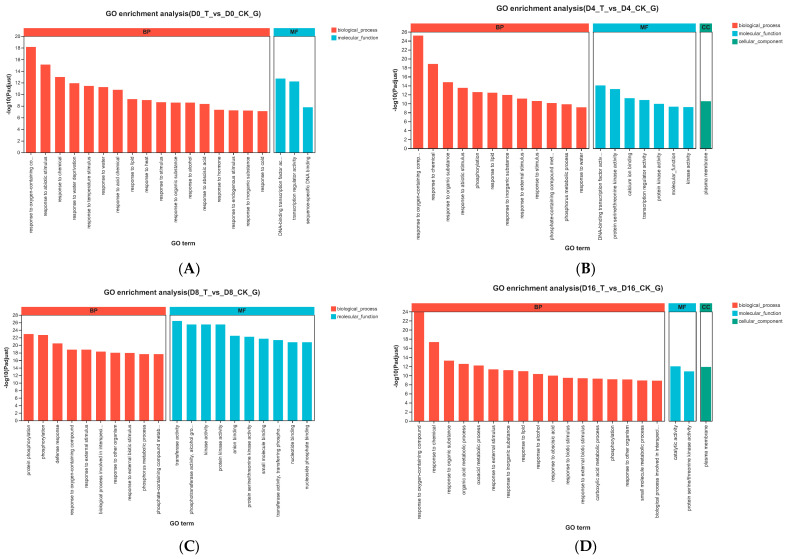
GO functional enrichment analysis of DEGs at 0 d (**A**), 4 d (**B**), 8 d (**C**) and 16 d (**D**).

GO functional analysis showed that the DEGs were mainly annotated to biological processes and molecular functions at 0 d and 8 d (Figure 4A,C). DEGs in the biological process categories were mainly associated with response to many kinds of conditions, such as oxygen-containing compounds, hormones, abiotic stimulus, etc. It was found that ‘response to oxygen-containing compound’ was the most enriched annotation at 0 d, 4 d and 16 d (Figure 4A,B,D). Functional enrichment to the ‘phosphorylation’ of DEGs came out at 4 d, which was the top five enriched annotations of biological processes (Figure 4B). ‘Protein phosphorylation, phosphorylation and defense response’ were the top three enriched biological process functions successively among DEGs at 8 d, while the first and the third function had not come out before (Figure 4C). Additionally, it was found that the enrichment of DEGs to ‘molecular function’ increased until 8 d and then declined at 16 d which meant more catalytic activity and binding function were executed. Compared with 0 d and 8 d, there was a little change in 4 d and 16 d, that is, the DEGs were also annotated to cellular components (Figure 4B,D). The only subgroup of the cellular component was the plasma membrane.

### 3.4. KEGG Enrichment Analysis of DEGs

**Figure 5:**
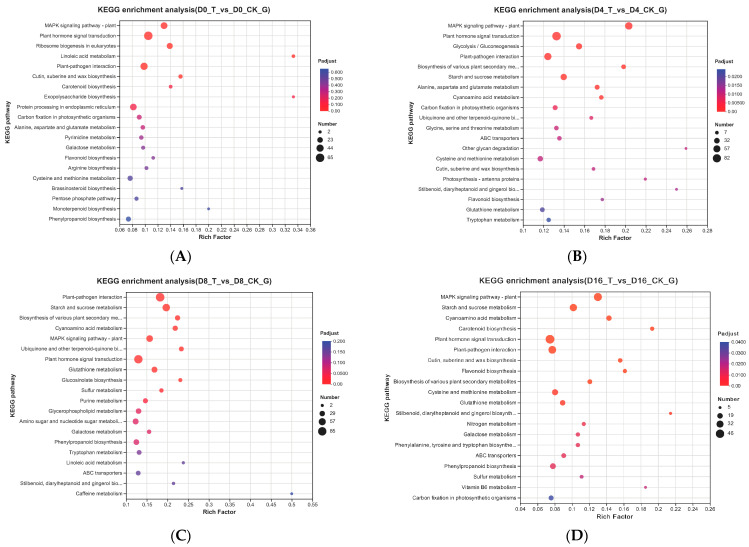
KEGG pathway enrichment analysis of DEGs at 0 d (**A**), 4 d (**B**), 8 d (**C**) and 16 d (**D**).

The 20 most enriched KEGG pathways of every storage time are shown in Figure 5. At the 0 d time point, DEGs were significantly enriched in the pathways of the MAPK signaling pathway, plant hormone signal transduction, ribosome biogenesis in eukaryotes, linoleic acid metabolism, plant–pathogen interaction and cutin, suberine and wax biosynthesis (Figure 5A), while at the 4 d time point, DEGs were significantly enriched in all the 20 pathways, and the top 5 enriched pathways were the MAPK signaling pathway, plant hormone signal transduction, glycolysis/gluconeogenesis, plant–pathogen interaction and the biosynthesis of various plant secondary metabolites (Figure 5B). At 8 d, the top 5 enriched pathways were plant–pathogen interaction, starch and sucrose metabolism, biosynthesis of various plant secondary metabolites, cyanoamino acid metabolism and the MAPK signaling pathway (Figure 5C). At 16 d, the top 5 enriched pathways were MAPK signaling pathway, starch and sucrose metabolism, cyanoamino acid metabolism, carotenoid biosynthesis and plant hormone signal transduction (Figure 5D). The analysis revealed that the MAPK signaling pathway and plant hormone signal transduction both exhibited significantly at every storage time.

### 3.5. DEGs Involved in Ethylene-Related Response

As DEGs were significantly enriched in the MAPK signaling pathway, plant hormone signal transduction and other pathways that might be related to plant ethylene, a further study of DEGs related to ethylene was carried out.

About 20 genes were screened out based on GO enrichment analyses (Table 2). All of them showed significant differences in expression between the CK and T groups at 4 d, and the value of Log_2_FC of 4 d is shown in Table 2. Among them, three genes were down-regulated, including *BolC5t34953H* (a probable *NAC*), *BolC1t05767H* (a probable *NAC*) and *BolC2t06548H* (a probable *ERF113*). The former two genes were annotated as a ‘positive regulation of ethylene biosynthetic process’. However, they were down-regulated in ClO_2_-treated samples, which might suggest that ClO_2_ treatment could delay the ethylene biosynthetic process. The latter one was a member of the *ERF* family, and it might negatively control ethylene biosynthesis, while the other 8 *ERF* and 2 probable *ERF* genes (*BolC1t01080H*, *BolC6t38599H*, *BolC4t28768H*, *BolC3t17122H*, *BolC8t48489H*, *BolC3t14461H*, *BolC7t43635H*, *BolC9t55823H*, *BolC3t18401H* and *BolC9t53913H*) behaved oppositely. *BolC1t04038H* (*ETR*), *BolC2t09157H* (*MKK9*), *BolC3t12569H* (*MPK4*) and *BolC9t54716H* (*Rboh*) were all involved in the MAPK signaling pathway, and they need further comprehensive analysis. The last three genes are new in KEGG database and need further excavation.

**Figure 6:**
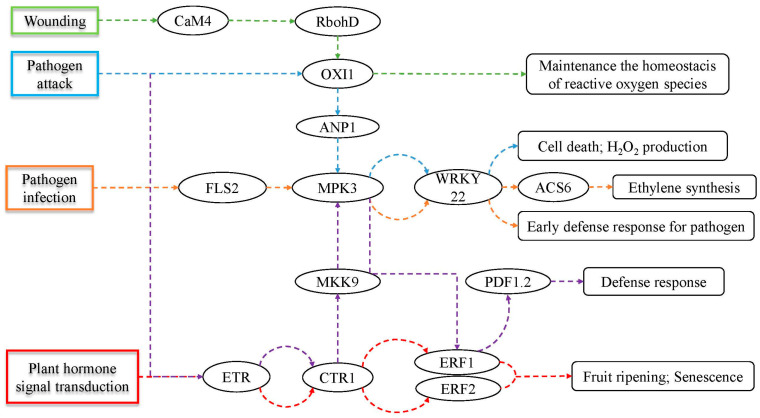
Up-regulated DEGs screened from ethylene-related pathways. Different color represents different pathway.

**Figure 7:**
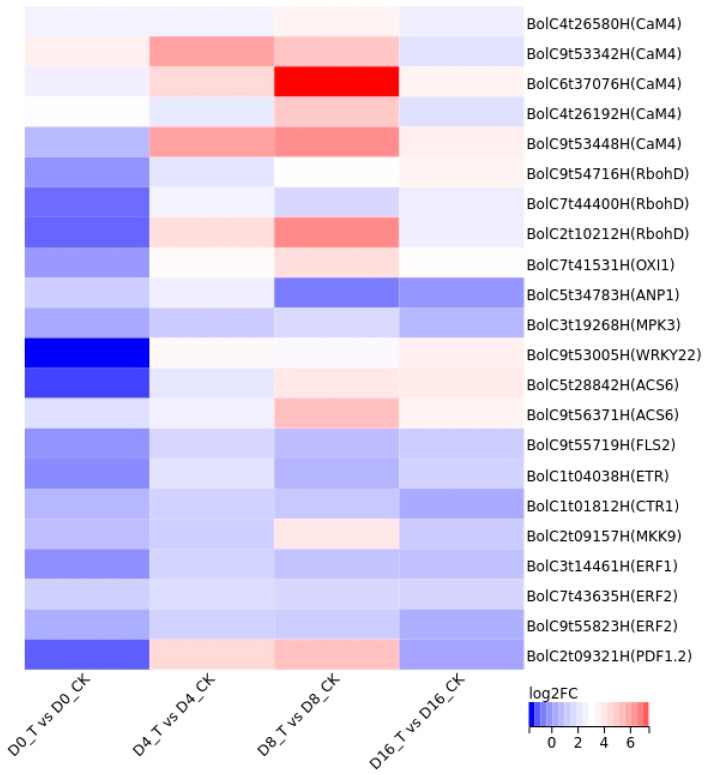
Expression analysis of DEGs involved in ethylene-related pathways.

A total of 22 genes were found up-regulated in ethylene-related signaling pathways, including the MAPK signaling pathway and plant hormone signal transduction, based on KEGG enrichment analyses (Figure 6). Except for *FLS2* (*BolC9t55719H*), *ETR*, *CTR1* (*BolC1t01812H*), *ERF1* and *ANP1* (*BolC5t34783H*), which were up-regulated only at 4 d, the other genes were up-regulated both at 4 d and 8 d (Figure 7).

As samples were fresh-cut products, the wounding branch of the MAPK pathway was initiated. In group T, *CaM4* (*BolC4t26580H*, *BolC9t53342H*, *BolC6t37076H*, *BolC4t26192H* and *BolC9t53448H*), *RbohD* (*BolC9t54716H*, *BolC7t44400H* and *BolC2t10212H*) and *OXI1* (*BolC7t41531H*) were up-regulated significantly for maintaining the homeostasis of ROS. *OXI1* was also involved in the pathogen attack branch of the MAPK pathway, and the *WRKY22* (*BolC9t53005H*) at the end of the branch was up-regulated for the defense of cell death and H_2_O_2_ production. *ETR* and *CTR1* were up-regulated in the pathogen attack branch by ClO_2_ treatment. The difference in *ETR* between CK and T was significantly increased at 4 d and 16 d, while the difference in *CTR1* was only significantly increased at 4 d (Figure 7). Finally, the last downstream gene *PDF1.2* (*BolC2t09321H*) was up-regulated by *ERF1* for defense response. Besides *ETR* and *CTR1*, *ERF1* and *ERF2* (*BolC7t43635H*, *BolC9t55823H*) that are involved in plant ethylene signal transduction were also up-regulated in the T group. For the pathogen infection branch, *FLS2*, *MPK3* (*BolC3t19268H*), *WRKY22* and *ACS6* (*BolC5t28842H*, *BolC9t56371H*) were found up-regulated for early defense response to pathogen and ethylene synthesis.

## 4. Discussion

Fresh-cut vegetables provide not only nutrients but also great convenience for cooking. However, they face a more arduous storage process than fresh products because of mechanical injury. Many reports have explored the antimicrobial mechanism of ClO_2_ and its effect on the postharvest management of horticultural products, e.g., spinach, cherry tomato [24,25,26,27,28]. Besides previous research from the physiological perspective, research is increasingly focusing on the molecular mechanism of how ClO_2_ acts in postharvest management.

In our study, the transcriptome method was used to investigate ethylene-related gene regulation in fresh-cut cauliflower florets under ClO_2_ conditions during storage. An RNA-seq analysis revealed that 2875, 3500, 4582 and 1906 DEGs were regulated at 0 d, 4 d, 8 d and 16 d, respectively (Figure 2). The Venn diagram of DEGs showed that 183 genes were existent at every storage time among all the DEGs (Figure 3). Then, genes related with ethylene were screened out based on GO and KEGG enrichment analyses.

According to RNA-seq, five and three members of *CaM4* and *RbohD* gene families are DEGs, respectively. *CaM* plays a critical role in Ca^2+^-modulated signaling processes and helps plants adapt to abiotic stress, while *RBOH* plays an important role in ROS production [29,30]. *RbohD* is mainly responsible for stress-induced ROS burst, while *RbohF*, up-regulated by *CaM4*, is responsible for the transient accumulation of superoxide in Arabidopsis [31]. *PpRbohE* is also up-regulated in trehalose-treated peach fruit in the ROS signaling pathway for resistance against chilling stress [32]. Similarly, the up-regulation of *CaM4* and *RbohD* in ClO_2_-treated cauliflower might be responsible for the maintenance of the homeostasis of ROS as being fresh-cut is a kind of abiotic stress and would inevitably lead to the production of ROS.

*OXI1* was reported to be activated by light, and it could enhance plant immunity through regulating responses and programmed cell death (PCD) [33,34]. In this study, *OXI1* was located on the junction of wounding and pathogen attack cascade. It was up-regulated and might induce the downstream gene *ANP1* that plays an important role in tomato and eggplant to mitigate the infection of *Tuta absoluta* [35]. *FLS2* that is up-regulated in pathogen infection cascade in ClO_2_-treated cauliflower is a receptor for flg22 and indirectly affecting the immunity of Arabidopsis [36]. Similar results and more detailed interactions have been reported in strawberry and rice [37,38].

The *WRKY* family is one of the largest transcript factor families in higher plants. *WRKY22-like*, *WRKY33* and *WRKY30* were down-regulated by high-oxygen-modified atmospheric packaging treatment on fresh-cut broccoli for mitigating oxidative damage from ROS accumulation [39]. Oppositely, *PpWRKY40*, *PpWRKY45*, *PpWRKY69* and *PpWRKY71* were up-regulated by trehalose treatment on peach fruit for promoting cold resistance; meanwhile, ROS-mediated antioxidant capability was promoted to eliminate excessive ROS production [32]. Our results showed that *WRKY22* was up-regulated by ClO_2_ treatment. Considering the pathway in which *WRKY22* is involved, it might function as an early defense response for pathogens. On the other hand, *WRKY22* might function to induce ethylene biosynthesis by activating *ACS6* expression (pathogen infection cascade) and promoting H_2_O_2_ accumulation (pathogen attack cascade). A similar function has been reported in carnation and oilseed rape [40,41]. *DcWRKY33* activated *DcACS1* and promoted petal senescence of fresh-cut carnation [40]. *BnaWGR1* activated *RbohD* and *RbohF* through binding to their promoters for the accumulation of H_2_O_2_, MDA and accelerated leaf senescence in oilseed rape [41].

As mentioned above, *ACS6*, located downstream of *WRKY22*, was up-regulated in ClO_2_-treated cauliflower for ethylene biosynthesis in response to pathogen infection stress. However, *LeACS2* and *LeACS4* were down-regulated by ClO_2_ treatment for the suppression of ethylene biosynthesis to delay the ripening of postharvest tomato [42]. Similar results were reported in ready-to-eat broccoli and fresh-cut ‘Hami’ melon by ClO_2_ treatment [18,43]. Different results might suggest the different roles of ethylene in biography processes.

Both *NACs* and *ERFs* are large transcript factor families like *WRKYs*. *SNAC4* could positively regulate ethylene synthesis by activating *SACS8* to promote tomato fruit ripening [44]. *DkNAC9* interacts with *DkERF8/16* to activate *DkEGase1*, and *DkERF18* activates *DkACS2*, both of which lead to persimmon fruit softening [45,46]. Herein, *BolC5t34953H*, *BolC1t05767H* and *BolC2t06548H* were all significantly down-regulated by ClO_2_ treatment (Table 2, Figure 7), which suggests that they may negatively regulate ethylene biosynthesis. Furthermore, *ETR* and *CTR1*, two negative regulators of ethylene response, were up-regulated by ClO_2_ treatment [47,48]. In summary, these five genes might be responsible for the suppression of ethylene biosynthesis.

Wang et al. reported the bidirectional regulation mechanisms of ethylene biosynthesis, where *ACS2* and *ACS6* were up-regulated by *MPK3* and *MPK6* in Arabidopsis to induce ethylene biosynthesis for pathogen defense, while *ERF1A* was triggered by *MPK3* and *MPK6* for the negative-feedback regulation of ethylene biosynthesis [49]. In our study, most genes including *MPK3* and *ACS6* (except for *ETR* and *CTR1*) up-regulated by ClO_2_ treatment might be responsible for inducing ethylene biosynthesis for pathogen defense and wounding defense. *BolC5t34953H*, *BolC1t05767H* and *BolC2t06548H* and *ETR* and *CTIR1* regulated by ClO_2_ treatment might be responsible for the suppression of ethylene biosynthesis. A similar mechanism might be found where genes regulate ethylene biosynthesis for both positive and negative sides and eventually delay the senescence of ClO_2_-treated fresh-cut cauliflower florets. However, more evidence should be found.

## 5. Conclusions

In this study, transcriptome analysis was conducted on ClO_2_-treated fresh-cut cauliflower florets. DEGs related to ethylene biosynthesis and ethylene signal transduction were screened and analyzed. Results showed that ethylene-related genes and pathways were involved in stress response in fresh-cut cauliflower florets during postharvest low-temperature storage. The up-regulation of genes including *OXI1*, *MPK3*, *WRKY22* and *ERF1* was a response to wounding, pathogen attack or infection defense. *ETR*, *CTR1*, *BolC5t34953H*, *BolC1t05767H* and *BolC2t06548H* involved in the ethylene signal transduction pathway might work as negative regulators for ethylene signal transduction. The two-side regulation showed that ethylene-related genes and pathways were involved in ClO_2_ treatment, which might enhance stress resistance and have a negative feedback mechanism for food preservation.

## Figures and Tables

**Table 1:** Statistics of RNA-Seq quality in cauliflower.

Sample_ID	Clean Reads	% > Q30	GC Content	Mapped Reads	Multiple Mapped Reads	Unique Mapped Reads
D0_CK_1	48256708	96.52	46.55%	90.45%	4.75%	85.7%
D0_CK_2	47668452	96.65	46.4%	90.34%	4.33%	86.01%
D0_CK_3	49227078	96.67	46.67%	90.48%	4.23%	86.25%
D0_T_1	43902798	96.71	46.22%	90.2%	3.81%	86.39%
D0_T_2	52426856	96.75	46.21%	90.27%	4.49%	85.78%
D0_T_3	55188122	96.63	46.17%	90.19%	4.51%	85.68%
D4_CK_1	57183564	96.56	46.18%	89.8%	3.09%	86.71%
D4_CK_2	50121392	96.59	46.15%	89.76%	2.95%	86.8%
D4_CK_3	47448964	96.62	46.09%	89.84%	3.29%	86.55%
D4_T_1	51790312	96.53	46.39%	90.15%	4.36%	85.79%
D4_T_2	55035510	96.61	46.29%	90.0%	4.49%	85.51%
D4_T_3	46029862	96.42	46.26%	90.07%	4.16%	85.92%
D8_CK_1	56371558	96.68	46.14%	89.67%	3.34%	86.33%
D8_CK_2	46888498	96.61	45.98%	89.7%	3.1%	86.6%
D8_CK_3	47708112	96.54	46.24%	89.66%	3.26%	86.39%
D8_T_1	52000634	96.65	46.38%	90.13%	4.17%	85.96%
D8_T_2	49258952	96.63	46.19%	90.11%	4.08%	86.03%
D8_T_3	48839766	96.7	46.19%	90.24%	4.06%	86.17%
D16_CK_1	55581818	96.53	46.16%	89.7%	2.99%	86.7%
D16_CK_2	47357874	96.62	46.11%	89.67%	3.31%	86.36%
D16_CK_3	59152866	96.7	46.08%	89.79%	3.46%	86.33%
D16_T_1	52493840	96.54	46.06%	89.88%	3.17%	86.71%
D16_T_2	50458528	96.5	46%	89.73%	3.3%	86.42%
D16_T_3	54911890	96.69	46.17%	89.55%	3.23%	86.33%

At each time point, there were three biological replicates from every single individual cauliflower floret. Each sample represented a biological replicate individually to form a unique library separately. CK: control; T: ClO_2_ treatment. D0_CK and D0_T represented samples at 0 day after storage. D4_CK and D4_T represented samples at 4 days after storage, and so on.

**Table 2:** Ethylene-related DEGs screened out based on GO enrichment analyses.

Gene ID	Gene Description/Annotation	Log_2_FC (4 d)
*BolC5t34953H*	probable *NAC*; positive regulation of ethylene biosynthetic process	−2.24
*BolC1t05767H*	probable *NAC*; positive regulation of ethylene biosynthetic process	−1.64
*BolC2t06548H*	probable ethylene-responsive transcription factor *ERF113*	−1.44
*BolC1t01080H*	ethylene-responsive transcription factor 1A	2.36
*BolC6t38599H*	ethylene-responsive transcription factor *ERF073*	4.29
*BolC4t28768H*	ethylene-responsive transcription factor ERF071	6.08
*BolC3t17122H*	ethylene-responsive transcription factor *RAP2-3*	1.14
*BolC8t48489H*	ethylene-responsive transcription factor 1A	3.00
*BolC3t14461H*	ethylene-responsive transcription factor 1	1.29
*BolC7t43635H*	ethylene-responsive transcription factor 2	1.60
*BolC9t55823H*	ethylene-responsive transcription factor 2	1.23
*BolC3t18401H*	probable ethylene-responsive transcription factor	1.83
*BolC9t53913H*	probable ethylene-responsive transcription factor	7.66
*BolC1t04038H*	Ethylene receptor (*ETR*)	1.81
*BolC2t09157H*	Mitogen-activated protein kinase kinase 9 (*MKK9*)	1.14
*BolC3t12569H*	MPK4; ethylene-dependent systemic resistance	3.10
*BolC9t54716H*	Receptor burst oxidase homolog (*Rboh*)	1.87
*BolC5t34361H*	ethylene-activated signaling pathway	1.11
*BolC9t58400H*	ethylene-activated signaling pathway	5.12
*BolC6t39722H*	ethylene-activated signaling pathway	4.63

## Data Availability

Data are contained within the article. The original contributions presented in this study are included in the article. Further inquiries can be directed to the corresponding author.
